# Special Issue “Viral-Induced Inflammation”

**DOI:** 10.3390/v16040588

**Published:** 2024-04-11

**Authors:** Simone de Araújo, Luciana P. Tavares, Vivian Vasconcelos Costa

**Affiliations:** 1 Department of Morphology, Institute of Biological Sciences, Federal University of Minas Gerais, Belo Horizonte 31270-901, Brazil; simonedearaujo@ufmg.br; 2 Pulmonary and Critical Care Medicine Division, Department of Medicine, Brigham and Women’s Hospital and Harvard Medical School, Boston, MA 02115, USA; lupt26@gmail.com

Inflammation is a protective host response essential for controlling viral replication and promoting tissue repair. However, uncontrolled or dysfunctional inflammation can exacerbate disease, leading to barrier dysfunction, organ failure, and ultimately, death. This Special Issue includes six original studies and one review focused on advancing our understanding of the pathogenesis of Venezuelan equine encephalitis virus (VEEV), Zika virus (ZIKV), and severe acute respiratory syndrome coronavirus 2 (SARS-CoV-2).

VEEV and ZIKV are arthropod-borne viruses transmitted through mosquito bites. In this Special Issue, we gained insights into the inflammation biomarkers of VEEV infection severity. Specifically, TNF, CCL2, CCL5, and leukocyte infiltration were shown as strong predictors of neuropathology induced by VEEV in mice (https://doi.org/10.3390/v15061307, accessed on 22 March 2024). Similarly, systemic inflammation, a “cytokine storm”, was reported to be associated with mortality in critically ill patients with COVID-19. Conversely, a robust, localized inflammatory response in the lungs was shown to be associated with better survival outcomes during SARS-CoV-2 infection (https://doi.org/10.3390/v15081704, accessed on 22 March 2024). Interestingly, a study by Hasan et al. suggested that antibodies for the SARS-CoV-2 N protein can contribute to exaggerated inflammation in COVID-19 by increasing the levels of IL-6 production from macrophages (https://doi.org/10.3390/v15102018, accessed on 22 March 2024). Building on pharmacological strategies to overcome COVID-19 disease, Pereira et al. demonstrated that inhibiting the 5-Lipoxygenase enzyme protected mice from severe lung disease induced by betacoronavirus infection without compromising the hosts’ ability to control virus replication (https://doi.org/10.3390/v15102049, accessed on 22 March 2024). Additionally, a study by Campolina-Silva et al. provided evidence for the protective role of vitamin D in decreasing viral loads and inflammation caused by both SARS-CoV-2 and murine coronaviruses (https://doi.org/10.3390/v15122434, accessed on 22 March 2024). A diet rich in vitamin D protected the mice from acute respiratory distress and systemic complications caused by these viruses, emphasizing that vitamin D is a key regulator of hosts’ responses to betacoronaviruses (https://doi.org/10.3390/v15122434, accessed on 22 March 2024). Lastly, a review by Len et al. underscored the significance of the early rise in myeloid-derived suppressor cells (MDSCs) in potentially mediating the disease progression of severe COVID-19 (https://doi.org/10.3390/v16010027, accessed on 22 March 2024). The authors reviewed the immunosuppressive actions of MDSCs derived from COVID-19 patients on T cells and discussed the potential therapeutic strategies targeting these cells to reduce the risk and severity of COVID-19 (https://doi.org/10.3390/v16010027, accessed on 22 March 2024).

In the context of ZIKV infection, we also learned that significant differences exist in microglial cell activation, viral replication, and the inflammatory and antiviral responses between ZIKV strains originating from African (ZIKVMR766) and Asian (ZIKVPE243) lineages (https://doi.org/10.3390/v15061250, accessed on 22 March 2024). The African strain induces more cell activation, a worse inflammatory response, and the decreased expression of certain antiviral factors during the early infection stages. Conversely, the Asian strain prompts notably increased levels of PPAR-γ and reduced levels of pro-inflammatory cytokines IL-6 and TNF. These findings are essential for understanding the pathogenesis of the diseases related to ZIKV and for developing therapeutic strategies that leverage innate immunity to effectively manage ZIKV infection (https://doi.org/10.3390/v15061250, accessed on 22 March 2024).

Finally, we extend our gratitude to all the authors for their invaluable contributions to this Special Issue. It has been a rewarding experience to engage with and learn from them. The publication of these studies undoubtedly aids in understanding the complexity of the dual role of inflammation in viral diseases and supports scientific development in the search for therapeutic strategies focused on the host to mitigate the viral disease burden.

